# A 13-Year Retrospective Study of Disease Patterns of Adenoviral Keratoconjunctivitis at a Tertiary Hospital in Switzerland (2012–2025)

**DOI:** 10.3390/microorganisms14102397

**Published:** 2026-10-20

**Authors:** Maximilian Jacobi, Anahita Bajka, Muntadher Al Karam, Daniel Barthelmes, Irene Voellmy, Michael Huber, Sadiq Said, Frank Blaser

**Affiliations:** 1 Department of Ophthalmology, Hospital of Zurich, University of Zurich, 8091 Zurich, Switzerland; 2 Talacker Augenzentrum Zürich, 8001 Zurich, Switzerland; 3 Institute of Medical Virology, University of Zurich, 8057 Zurich, Switzerland; 4 Augenklinik Wettingen, 5430 Wettingen, Switzerland

**Keywords:** adenovirus keratoconjunctivitis, keratoconjunctivitis epidemica, epidemic keratoconjunctivitis, hygiene measures, COVID pandemic, AdenoPlus^®^ test, PCR test

## Abstract

To report epidemiological patterns of adenoviral keratoconjunctivitis at a tertiary care hospital in Switzerland from 2012 until 2025, particularly comparing incidence rates during the COVID-19 pandemic. A single-centre, retrospective, observational study including all patients diagnosed with adenoviral keratoconjunctivitis from May 2012 until March 2025, describing patients’ demographics (gender, age), analysing and comparing incidence rates prior, during and after the COVID-19 pandemic. All cases were diagnosed with PCR testing and/or the AdenoPlus^®^ test. During the COVID-19 pandemic, incidence rates of Adenovirus keratoconjunctivitis dropped from a mean number of 1.15 (SD ± 1.27, range: [0–6]) cases per month down to 0.57 (SD ± 0.79, range: [0–2]) during the COVID-19 pandemic. Overall incidence rates showed seasonal fluctuations, with most infections occurring in autumn months. Furthermore, demographic analysis revealed a significant gender disparity with higher male incidence (male:female ratio 1.39:1). During the quarantine measures of the COVID-19 pandemic, incidence of laboratory-confirmed Adenovirus keratoconjunctivitis was significantly reduced, which might be associated with social distancing and increased hygiene measures. After cessation of quarantine measures, incidence rates returned to pre-pandemic levels. This study provides insight into epidemiological patterns of Adenovirus keratoconjunctivitis while emphasising the importance of hygiene measures in preventing diseases.

## 1. Introduction

Adenoviral keratoconjunctivitis is among the most common viral infections of the ocular surface and is a frequent reason for ophthalmologic consultation worldwide. The disease affects all age groups and is associated with considerable morbidity, temporary to permanent visual impairment, and increased healthcare utilisation [1]. Adenoviruses belong to the family *Adenoviridae* and are non-enveloped, double-stranded DNA viruses classified within the genus *Mastadenovirus* [2]. The lack of a viral envelope makes Adenoviruses relatively insensitive to alcohol-based disinfectants and, therefore, exhibits high contagiousness [1]. Transmission occurs primarily through direct contact with infected ocular secretions or contaminated fomites. Respiratory droplets have occasionally been described as an additional route of spread [3].

**Figure 1:**
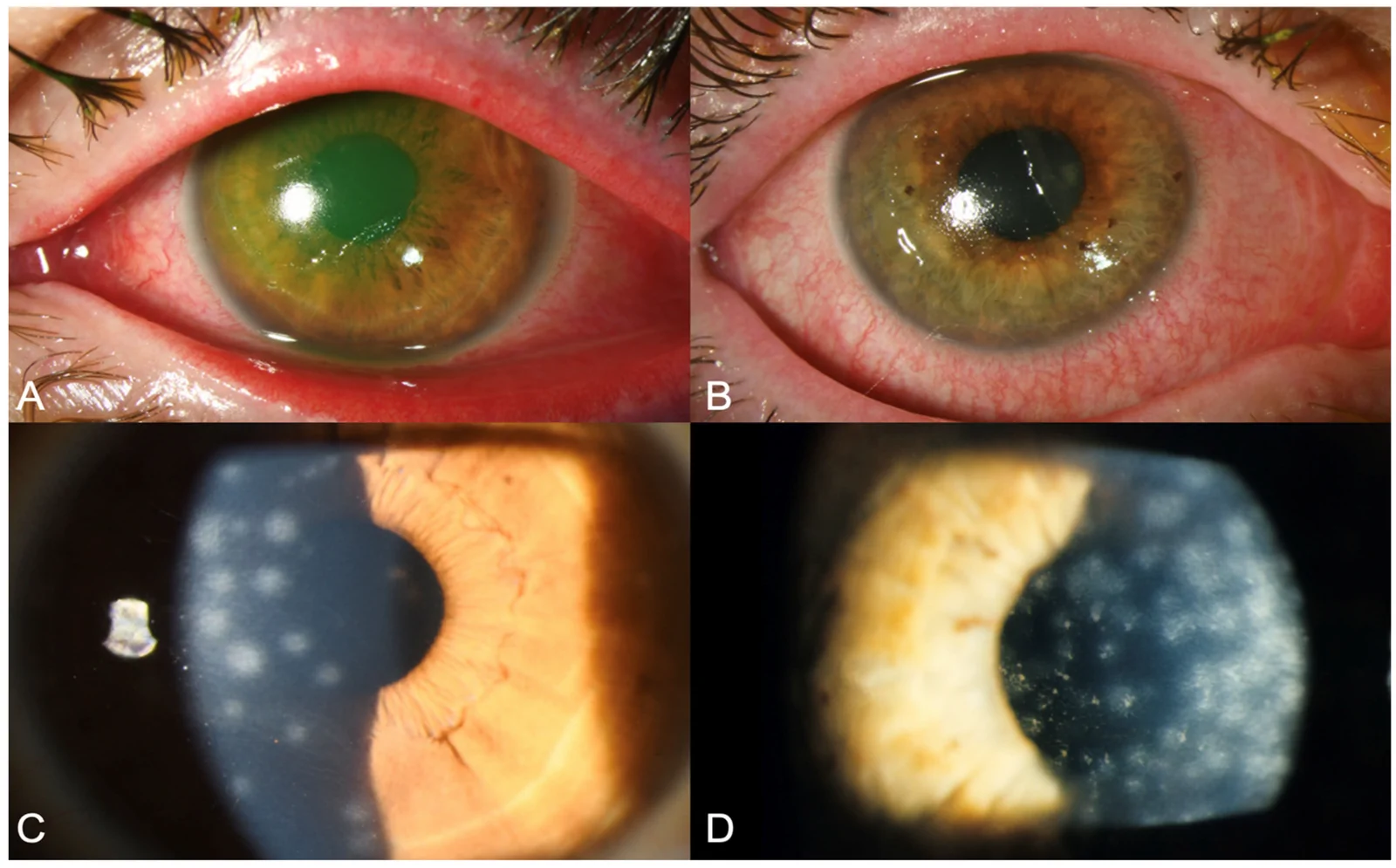
Clinical findings in Adenovirus keratoconjunctivitis. (**A**,**B**) show conjunctival hyperemia, including palpebral inflammation. The corneal epithelium is roughened and irregular, showing a corneal erosion as well. After the initial infection, subepithelial infiltrates (called nummuli) can manifest as an immune response, as shown in (**C**,**D**).

Ocular infection typically presents with acute conjunctival inflammation characterised by redness, tearing, photophobia and foreign-body sensation. Although most cases are self-limiting, complications may occur and lead to prolonged healing time [4]. Clinically, adenoviral keratoconjunctivitis often follows a biphasic course. After an incubation period of about 5 to 12 days, patients typically present with an acute conjunctival phase characterised by unilateral or bilateral conjunctival injection, chemosis, ocular discomfort, pruritus, and watery discharge (see Figure 1) [5]. Symptoms often begin in one eye before spreading to the other. Bilateral involvement has been reported in up to 70% of cases [6]. Patients commonly report photophobia and foreign-body sensation, some even develop eyelid edema. A characteristic finding in more severe cases is the presence of conjunctival pseudomembranes or true membranes, which adhere to the conjunctival surface and typically bleed upon removal [7]. Corneal involvement may develop several days after conjunctival symptoms begin. During this stage, multiple grey-white subepithelial infiltrates can appear in the anterior corneal stroma. These lesions represent immune-mediated inflammatory reactions rather than active viral replication and may cause glare, photophobia, and mild up to severe permanent reduction in visual acuity if scarring results. Although the disease usually resolves spontaneously, these infiltrates may persist for months and can lead to prolonged or permanent visual complaints [8]. Additional complications, including symblepharon formation or toxic keratopathy related to topical treatments, may further prolong recovery and finally affect quality of life [9].

Adenoviruses responsible for ocular disease belong predominantly to species D, with serotypes 8, 19, 37, 54, and 56 most commonly implicated in epidemic keratoconjunctivitis [10]. However, several additional serotypes—including 2, 3, 4, 5, 7, 10, 11, 21, 22, 29, and 34—have also been associated with adenoviral keratoconjunctivitis [11,12].

From an epidemiological perspective, adenoviral keratoconjunctivitis is among the leading causes of infectious conjunctivitis worldwide [13]. The infection occurs globally and shows considerable variation across geographic regions, climate conditions, and population density [14]. Although cases arise throughout the year, several studies report seasonal peaks, particularly during late spring and early summer [15,16,17]. In temperate climates, the infection typically exhibits endemic circulation [18]. National surveillance data from multiple countries in Europe have reported periodic surges in Adenovirus keratoconjunctivitis case numbers over the past two decades, which can be partially attributed to improved outbreak reporting and the wider availability of molecular diagnostic techniques such as PCR [19,20]. Although clinicians often diagnose adenoviral keratoconjunctivitis based on characteristic clinical findings, laboratory confirmation such as polymerase chain reaction (PCR) can support diagnosis and facilitate epidemiological investigations [21,22,23,24,25].

Despite its high prevalence and public health impact, longitudinal data describing the temporal patterns of adenoviral keratoconjunctivitis in Switzerland remain limited. Better understanding of local disease trends may support clinical awareness, infection control strategies, as well as healthcare planning. In this retrospective chart review, we aimed to analyse the annual trends of adenoviral keratoconjunctivitis diagnosed at a tertiary ophthalmology centre in Switzerland over an extended observation period.

The COVID-19 pandemic of 2019 saw substantial quarantine measures put in place in Switzerland to limit the spread of the SARS-CoV-2 and ultimately save lives. These included obligatory wearing of medical face masks, social distancing, as well as increasing hygiene awareness. Although reductions in conjunctivitis during the COVID-19 pandemic have previously been reported, long-term data based specifically on laboratory-confirmed adenoviral keratoconjunctivitis remain limited. The present study therefore aimed to characterise temporal, seasonal, and demographic patterns of laboratory-confirmed adenoviral keratoconjunctivitis at a Swiss tertiary ophthalmology centre over a 13-year period and to assess changes in case frequency during and after the COVID-19 public health interventions.

## 2. Materials and Methods

We conducted a single-centre, retrospective observational study at the Department of Ophthalmology, University Hospital of Zurich in Switzerland. The study evaluated the incidence and annual patterns of laboratory-confirmed adenoviral keratoconjunctivitis between May 2012 to March 2025. Further, we analysed potential changes in epidemiological patterns during the COVID-19 pandemic. Therefore, we defined the pandemic period from 20 March 2020 to 16 February 2022, corresponding to the public health measures implemented by the Swiss Government during that period. The leading ethics committee waived formal ethical approval (BASEC number Req-2025-00917), as this study did not fall within the scope of the Human Research Act. Nevertheless, we handled all data according to Good Clinical Practice (GCP) guidelines.

### 2.1. Data Collection

We identified all patients who underwent laboratory testing for suspected adenoviral keratoconjunctivitis during the study period. At our institution, laboratory testing for Adenovirus is not routinely performed in all patients presenting with conjunctivitis but is obtained when adenoviral keratoconjunctivitis is clinically suspected. Only laboratory-confirmed adenoviral infections were included in the present analysis. Confirmation of adenoviral keratoconjunctivitis required a positive laboratory test result obtained by real-time polymerase chain reaction (PCR) or by the AdenoPlus^®^ Adenoviral Conjunctivitis Rapid Test (Rapid Pathogen Screening, Inc., Sarasota, FL, USA). AdenoPlus^®^ tests were used for diagnosis from July 2014 until January 2023. We restricted case inclusion to laboratory-confirmed infections to minimise diagnostic bias.

### 2.2. Adenovirus Detection by Real-Time PCR

Sample preparation and DNA extraction: Upon arrival at the laboratory, specimens were vortexed, swabs removed, and the remaining viral transport medium (VTM) centrifuged at 2000 rcf for 10 min. DNA extraction was performed from 200 µL of supernatant and eluted into 110 µL of elution buffer (Extraction Buffer 3), using easyMAG and EMAG^®^ nucleic acid extraction systems according to the manufacturer’s protocols (bioMérieux, Marcy l’Etoile, France). From 28 August 2024, the extraction protocol was modified to increase assay sensitivity by increasing specimen input volumes and reducing elution volumes as follows: depending on the number of analyses required, nucleic acid was extracted from 600, 400, or 300 µL of supernatant and was eluted into 60, 40, or 30 µL of elution buffer (Extraction Buffer 3), respectively. For PCR process control, all specimens were spiked with a standardised amount of phocid herpesvirus type 1 (PhHV) prior to extraction (European Virus Archive EVAG, Marseille, France, https://www.european-virus-archive.com, [26]).

DNA amplification: Real-time PCR was performed using in-house protocols with primers and TaqMan^®^ probes synthesised by Microsynth (Microsynth AG, Balgach, Switzerland). The following sequences of primers and probe for Adenovirus detection were used, generating a 132 bp amplicon of the Hexon gene: forward primer 5′-GCC ACG GTG GGG TTT CTA AAC TT-3′, reverse primer 5′-GCC CCA GTG GTC TTA CAT GCA CAT C-3′, probe 5′-FAM-TGC ACC AGA CCC GGG CTC AGG TAC TCC GA-BHQ1-3′ [27]. Until 28 August 2024, Adenovirus was detected using a singleplex assay, with an additional singleplex process control, generating an 89 bp amplicon of the human GAPDH gene (Homo sapiens glyceraldehyde-3-phosphate dehydrogenase). Sequences of primers and probe for GAPDH were as follows: forward primer 5′-CAA GGT CAT CCA TGA CAA CTT TG-3′, reverse primer 5′-GGC CAT CCA CAG TCT TCT GG-3′, probe 5′-VIC-ACC ACA GTC CAT GCC ATC ACT GCC A-TAMRA-3′ [28]. Each Adenovirus assay was run in duplicate to enhance sensitivity. Adenovirus and GAPDH mastermixes contained 900 nM of each primer and 200 nM of the respective probe in 25 µL of TaqMan Universal PCR MMIX II (Applied Biosystems by Thermo Fisher Scientific, Waltham, MA, USA), supplemented with 19 µL of nuclease-free water (AppliChem GmbH, Darmstadt, Germany). RT-PCR amplifications were performed using 45 µL of master mix and 5 µL of eluted DNA in a total volume of 50 µL. From 28 August 2024, GAPDH was replaced by phocid herpesvirus type 1 (PhHV) glycoprotein B as process control, by spiking all specimens with a standardised amount of PhHV prior to extraction. All assays were performed as duplex reactions with probes and primers targeting Adenovirus and PhHV, respectively. Primers and probes detecting PhHV were as follows: forward 5′-GGG CGA ATC ACA GAT TGA ATC-3′, reverse 5′-GCG GTT CCA AAC GTA CCA A-3′, probe 5′-VIC-TTT TTA TGT GTC CGC CAC CAT CTG GAT C-BHQ1-3′, generating an 89 bp amplicon [26]. Mastermixes included 780 nM Adenovirus primers, 50 nM PhHV forward primer, 200 nM PhHV reverse primer, 200 nM Adenovirus probe, and 100 nM PhHV probe in 25 µL of TaqMan Master Mix II, and 17.3 µL of nuclease-free water. Total reaction volume remained 50 µL but consisted of 40 µL of mastermix and 10 µL of eluted DNA. The modified protocol, which included a 10-fold DNA concentration during extraction and doubled volumes of eluted DNA per PCR-reaction, increased test sensitivity by approximately 10 fold, allowing elimination of assay duplicates. QuantStudio™ 3 Real-Time PCR System (Applied Biosystems, Singapore) was used for amplification with the following cycling condition: 2 min at 50 °C, 10 min at 95 °C, followed by 50 cycles of 95 °C for 15 s and 60 °C for 1 min. Each run included a negative control (NUCLISENS^®^ easyMAG^®^ Extraction Buffer 3, bioMérieux, Marcy-l’Étoile, France) and an Adenovirus-specific positive control, obtained from a NEQAS external quality control specimen (no. 2141/1990) containing Adenovirus type 3, cultured in A549 cells. Samples with cycle thresholds (Ct) of <38 were considered positive. Samples with Ct ≥ 38 were retested and only reported positive if confirmed. For duplicated assays, a single positive replicate was sufficient to be reported as positive. Process control signals (GAPDH or PhHV) had to be detected before cycle 33 to accept results and to ensure no loss of DNA or PCR inhibition during the extraction and amplification process.

### 2.3. AdenoPlus^®^ Adenoviral Conjunctivitis Rapid Test

The AdenoPlus^®^ Adenoviral Conjunctivitis Rapid Test is a point-of-care immunochromatographic assay designed to detect adenoviral hexon antigen in conjunctival samples [29]. The test kit consists of a sterile Dacron sampling collector, a buffer vial, and a test cassette.

Samples were obtained by gently swabbing the palpebral conjunctiva until the sampling tip was adequately saturated with conjunctival fluid. The collector was then inserted into the test cassette, and 1–2 mL of the supplied buffer solution was applied for approximately 20 s to facilitate antigen transfer. After an incubation period of 10 min, results were interpreted according to the manufacturer’s instructions.

The assay detects adenoviral hexon protein using labelled antibodies in a lateral-flow immunochromatographic format. Previous studies have reported a high specificity of approximately 98% and sensitivities ranging between 85% and 90% when compared with PCR [30]. However, sensitivity may vary considerably across clinical settings and negative results should therefore be interpreted cautiously [31].

### 2.4. Statistical Analyses

We applied descriptive statistics, presenting means with standard deviations and medians with minimum and maximum values for continuous variables and absolute numbers with percentages for categorical variables. When both PCR and AdenoPlus^®^ testing were performed simultaneously, the result was counted as a single positive case (accounting for one singular case, which was counted among the PCR results). Incidences of adenoviral keratoconjunctivitis were recorded by year and month. To visualise annual trends, we calculated a 6-month moving average to smooth short-term fluctuations. For analysis of seasonality, Seasons were defined using meteorological standards (Spring = March–May; Summer = June–August; Autumn = September–November; Winter = December–February) [32].

To compare monthly infection counts during the COVID-19 pandemic (March 2020–February 2022) with non-pandemic periods, we used the nonparametric Kruskal–Wallis test. Serial dependencies within the time series were evaluated using the Box–Ljung test. Analysis of demographical data (age, gender) as well as seasonal fluctuations were performed using χ^2^-Test and a Poisson generalised mixed effects model. All statistical analyses were performed using the R statistical software (version 4.5.1) environment.

## 3. Results

Reviewing all laboratory examinations for suspected adenoviral keratoconjunctivitis performed at our tertiary care centre between May 2012 and April 2025, we identified 747 test results. These included 557 PCR tests and 190 AdenoPlus^®^ tests between October 2014 and April 2023. Overall, 22.9% of PCR tests (*n* = 128) and 26.8% of AdenoPlus^®^ tests (*n* = 51) were positive for Adenovirus. Across all diagnostic modalities, 179 (23.9%) tests yielded positive results.

### 3.1. Demographic Analysis

Complete demographic data were analysed for all 179 confirmed cases. The median (IQR, range) age was 40.0 (31.0 to 54.0, 3.0 to 91.0) years. Age distribution peaked in the fourth and fifth decades of life, with 81 cases (45.25%) occurring in patients aged 30 to 49 years. Paediatric cases (<18 years) were uncommon, accounting for 4.5% of infections (*n* = 8). The cohort demonstrated a male predominance of 58.1% versus 41.9% (ratio 1.39:1), which was similar across both diagnostic modalities (Table 1). The gender difference was statistically significant over all tests performed (*p* = 0.0302).

### 3.2. Temporal Distribution of Laboratory-Confirmed Adenovirus Keratokonjunctivitis

Over the study period, a mean (SD) of 1.15 (1.27) laboratory-confirmed cases was identified per month (Table 2).

The baseline period from 1 May 2012 to 19 March 2020 covered 96 months and included 477 tests performed, including 331 PCR tests and 146 AdenoPlus^®^ tests. A total of 116 (24.3%) positive cases were recorded during the baseline period, with 24.4% (*n* = 81) PCR tests and 24.6% (*n* = 36) AdenoPlus^®^ tests being positive. This corresponded to a mean (SD) monthly incidence of 1.21 (1.29) cases. During this period, case numbers ranged from 0 to 6 infections per month.

During the COVID-19 pandemic (20 March 2020, to 16 February 2022), a total of 49 tests were performed over 23 months, including 20 PCR tests and 29 AdenoPlus^®^ tests. Among these, 26.5% (*n* = 13) were positive for Adenovirus, including 10.0% of PCR tests (*n* = 2) and 37.9% of AdenoPlus^®^ tests (*n* = 11).

**Figure 2:**
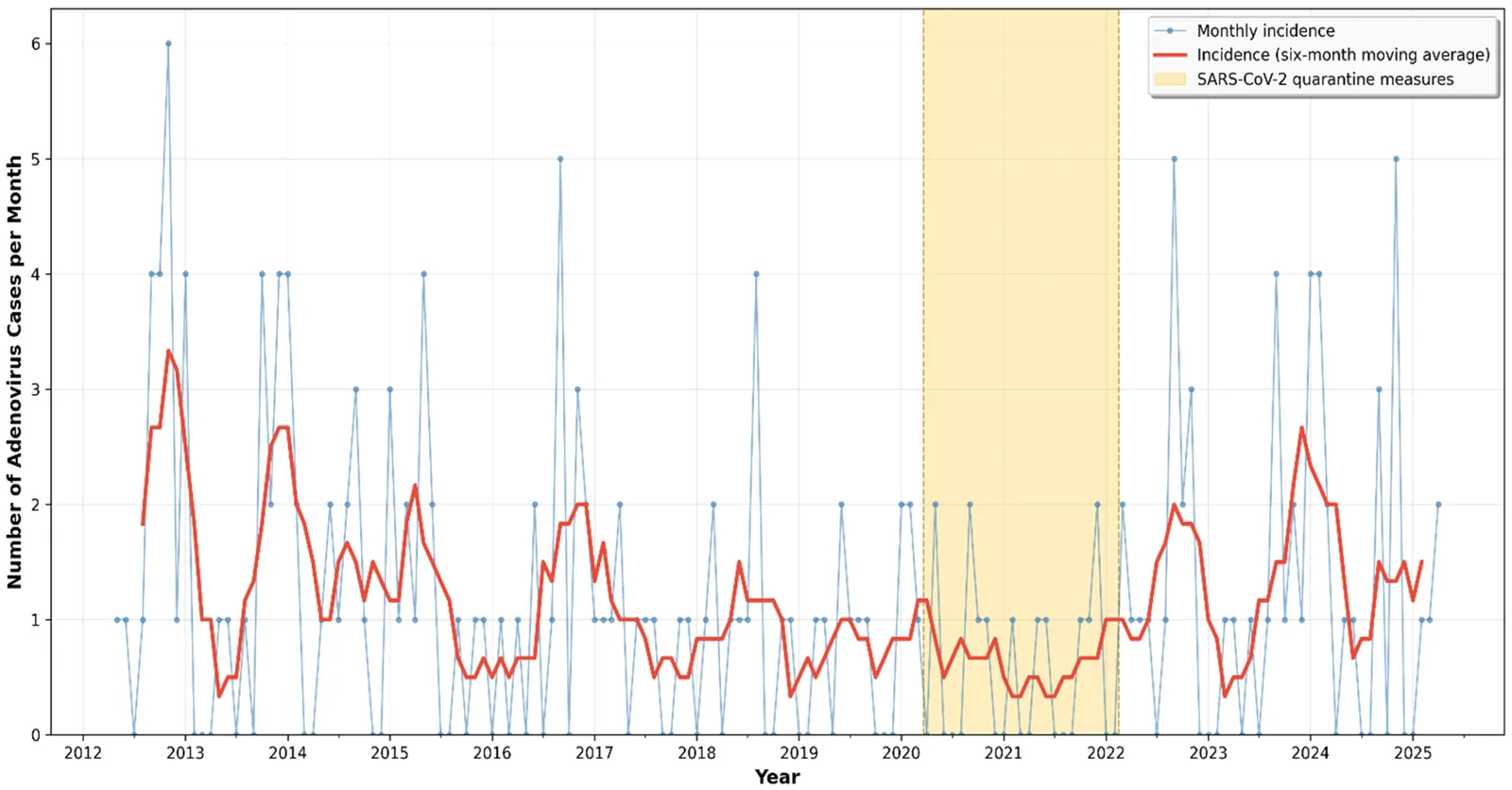
Rates of monthly incidence of adenoviral keratoconjunctivitis, using PCR or AdenoPlus^®^ test for diagnosis, in the months between May 2012 and April 2025 (blue line). The interval in yellow indicates the COVID-19 pandemic, corresponding to months 97–119. A 6-month moving average is displayed in red.

The mean (SD) monthly incidence rate showed a 54.7% reduction (*p* = 0.009) during the COVID-19 period compared with baseline conditions. The 6-month moving average confirmed this trend, showing a marked decline during the intervention period, followed by a gradual recovery (Figure 2).

In the post-COVID period (17 February 2022, to 30 March 2025), 221 tests were performed over 37 months, including 206 PCR tests and 15 AdenoPlus^®^ tests. We documented 50 positive cases over 37 months, with 21.8% (*n* = 45) of PCR tests and 33.3% (*n* = 5) of AdenoPlus^®^ tests being positive. The mean (SD) monthly incidence of 1.35 (±1.36) suggests a return to pre-pandemic case numbers, with incidence levels slightly exceeding those observed during the baseline period (Table 3). Kruskal–Wallis analysis showed no significant difference (*p* = 0.465) in rates of cases.

### 3.3. Seasonality of Adenovirus Keratoconjunctivitis

**Figure 3:**
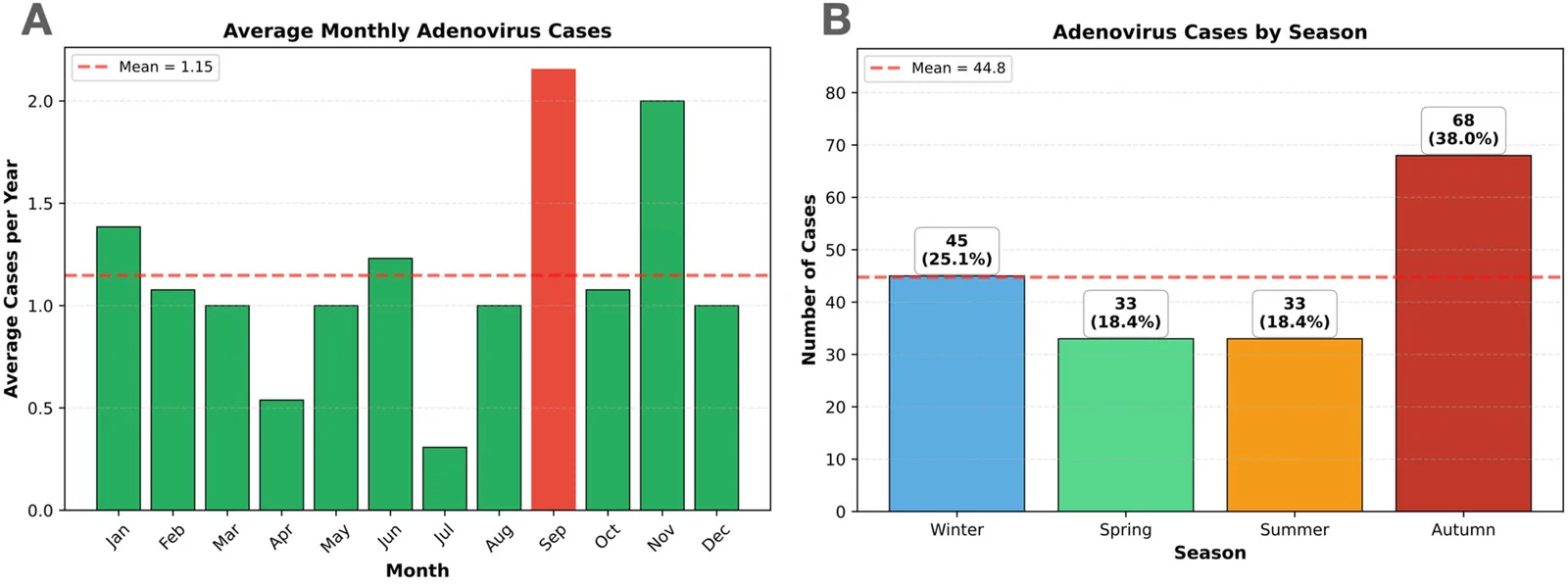
Seasonal incidence of Adenovirus keratoconjunctivitis. (**A**) Incidences by average number of cases per month (average monthly incidence overall shown by red line). The month with the highest average number of cases is shown in red. (**B**) Incidence by total number of cases per season (average incidence shown by red line). The data show a clear increase in cases during autumn (38% of all cases), peaking in September (2.15 cases/month).

Seasonality analysis revealed annual variations in Adenovirus keratoconjunctivitis incidence (Figure 3). A substantial peak was observed in autumn, with 38.0% of all cases occurring during autumn months. Analysis using a Poisson generalised mixed effects model showed the autumn peak being statistically significant compared to the other seasons (*p* = 0.027, 0.008 and 0.001 for winter, spring and summer, respectively). September exhibited the highest monthly incidence (*n* = 28, 15.6% of total cases), while July showed the lowest (*n* = 4, 2.2%).

## 4. Discussion

The present study documented a significant reduction in laboratory-confirmed Adenovirus keratoconjunctivitis during the period of enhanced hygiene measures associated with the COVID-19 pandemic in Switzerland. The observed reduction of 54.7% is both statistically significant (*p* = 0.009) and clinically relevant, indicating the potential effect of non-pharmaceutical interventions (isolation, increased hygiene measures, etc.) on the transmission of respiratory viruses. In line with other infectious diseases, the decreased incidence of this highly contagious disease can likely be attributed to increased hygiene measures, social distancing and increased awareness of sources for infection. It is important to remember that Adenoviruses are transmitted predominantly via contact transmission from fomites, while the SARS-CoV-2 is transmitted both by contact and airborne transmission. Thus, while the transmission of the two diseases does differ, both diseases would still be similarly affected by such hygiene measures, which aimed at limiting both contact and airborne transmission. Several studies have examined rates of Adenovirus keratoconjunctivitis during the COVID-19 pandemic [33,34,35,36]. Most studies found a decrease in incidence during the pandemic, with the number of cases rising again after quarantine measures were lifted [37,38]. Importantly, the existing studies of adenoviral keratoconjunctivitis during the pandemic generally determine positive Adenovirus infection using clinical parameters, not laboratory testing. Studies have shown parallel developments for other infectious diseases during the pandemic. For example, significant reductions in incidence rates could be seen for influenza [39], measles, pertussis and tuberculosis [40]. It has also been shown that many infectious diseases showed a substantial surge after the pandemic [41,42]. The documented post-pandemic surge in respiratory infections mirrors our observation of Adenovirus return to baseline, providing temporal validation across the disease spectrum.

Although viral conjunctivitis is frequently diagnosed clinically, symptoms such as conjunctival hyperaemia, irritation, and excessive tearing are nonspecific and may also occur in bacterial, allergic, chemical, or mechanical conjunctivitis. In the Department of Ophthalmology at the University Hospital of Zurich, laboratory confirmation is routinely performed in addition to the clinical diagnosis of adenoviral keratoconjunctivitis. Owing to the high transmissibility of adenoviral infections, virological confirmation is used to facilitate the timely implementation of appropriate infection control measures and, where applicable, the issuance of certificates of temporary work incapacity. Throughout the study period, polymerase chain reaction (PCR) served as the standard diagnostic method. For a limited period, particularly in healthcare workers, the AdenoPlus^®^ test was additionally used because of its shorter turnaround time. However, following its withdrawal from the market, this assay was no longer available and was consequently discontinued.

Restricting inclusion to laboratory-confirmed cases allowed a more reliable assessment of disease incidence. The relatively low absolute number of cases observed in this study reflects the hospital-based design and should not be interpreted as the population incidence of adenoviral keratoconjunctivitis in the Zurich region. As a tertiary referral centre, our institution represents only one component of regional ophthalmic care, while uncomplicated conjunctivitis is frequently managed in primary care, private ophthalmology practices, and other healthcare institutions. Furthermore, our analysis deliberately included only laboratory-confirmed cases. The principal value of the dataset, therefore, lies not in estimating population incidence, but in examining longitudinal changes within a consistently defined, laboratory-confirmed cohort over a 13-year period.

Overall, demographic analysis revealed a significant male predominance (58.1% versus 41.9%, 1.39:1). Several mechanisms may explain this pattern, including occupational exposure differences, behavioural factors affecting hygiene compliance, immunological differences, or healthcare-seeking behaviours. The age distribution was similar to comparable epidemiological studies [43].

The incidence rates further displayed a robust seasonal pattern, with 38% of cases in autumn months (χ^2^ = 18.25, *p* = 0.0004). This data matches with similar studies, which all show seasonal variability. However, the data are inconsistent regarding the incidence peak, with literature describing peaks in summer as well as in winter months [16,43]. In temperate climates, viral infectious diseases typically show peak infections in autumn and winter months [44]. It must be noted however, that these studies had been performed in different climatological environments than Switzerland. The consistent seasonal pattern across all 13 years of observation—including pre-, during, and post-pandemic phases—demonstrates robust epidemiology independent of pandemic-related artefacts.

The strength of this analysis lies in the long observation period of more than a decade and the use of a robust pre-pandemic baseline. Interrupted time series analysis is an established method for evaluating public health interventions and allows control for pre-existing trends. While several other sources have studied the effects of the pandemic on Adenovirus keratoconjunctivitis, they tend to rely on clinical diagnosis [33,34,35,36]. Laboratory confirmation of all cases via PCR or rapid antigen testing eliminates misclassification bias inherent in clinical-only diagnosis—especially important in Adenovirus keratoconjunctivitis, where clinical presentations can be diffuse and atypical.

Due to the retrospective design, testing bias must be considered as a potential confounder of the results and analysis. However, due to the high transmission rate of the infection and the associated need for strict hygiene measures, patients in our institute presenting with suspicion of Adenovirus keratoconjunctivitis consequently receive laboratory testing (AdenoPlus^®^ test, PCR testing or both). Therefore, it can be argued that the data are not likely to be heavily influenced by testing bias. It must be noted that the AdenoPlus^®^ test has slightly lower sensitivity than PCR in detecting Adenovirus (85–90% sensitivity compared to PCR), which might have erroneously underrepresented the true incidence rates during the time period from October 2014 to April 2023, including the totality of the COVID-19 interval [29,30]. During this time period, 50.5% of all tests were AdenoPlus^®^ (51 out of 101 test). The AdenoPlus^®^ test was performed during a majority of the overall as well as the pre-COVID-19 time intervals as well, limiting potential bias for the comparison of cases during and outside of the pandemic. However, since post-COVID-19 the testing was purely performed by PCR, there is a risk of underrepresenting the true case numbers in previous time intervals, potentially affecting comparison of pre-COVID19 and post-COVID19 case numbers. Even so, it seems unlikely for this to affect the finding that case numbers returned to pre-pandemic levels after quarantine measures were lifted. Specificity was similar between AdenoPlus^®^ tests and PCR tests (98% compared to PCR) and need not be realistically considered for potential error in data analysis.

In terms of the statistical analysis, the Ljung–Box Test showed independence of the serial data (*p* = 0.25). Larger lag dependencies are to be considered unrealistic and are not tested. Furthermore, it must be noted that measuring monthly incidence rates skews data in March 2020 and February 2022, which included both data within and beyond the COVID-19 pandemic.

These findings may offer further insight prevention of infections, especially in cases of localised Adenovirus epidemics [45,46,47], which can have severe impacts on quality of life, general health (especially in case of vulnerable patient populations such as neonates) and socioeconomic factors. This is particularly relevant for vulnerable populations such as immunocompromised patients or paediatric cohorts.

### Limitations

This study has several limitations that warrant consideration. First, the single-centre design limits generalisability to other healthcare settings with different patient populations and testing practices. Particularly during an exceptional societal event like the pandemic, patient behaviour cannot be relied upon to adhere to standard norms, causing the potential for significant bias in response to both the rate of Adenovirus infection as well as presentation at a clinic. Second, the observational nature precludes definitive causal inference, as multiple concurrent interventions during the pandemic period cannot be individually assessed. Third, potential detection bias exists since the decision to perform a test will be determined by clinical characteristics based on the clinician’s discretion, as well as due to evolving diagnostic testing priorities, with possible underrepresentation of Adenovirus cases during periods of intense SARS-CoV-2 surveillance. Fourth, with a relatively small absolute number of cases, specifically during the COVID-19 pandemic, statistical power is lowered.

Finally, because this was a hospital-based rather than population-based study, population incidence rates cannot be derived from our data. The absolute case numbers reflect only laboratory-confirmed infections presenting to our tertiary centre and likely substantially underestimate the total burden of adenoviral keratoconjunctivitis in the regional population.

## 5. Conclusions

This study provides meaningful insight into annual patterns of Adenovirus keratoconjunctivitis cases in Switzerland, covering a period of 13 years. Seasonality analysis, not previously analysed for Adenovirus keratoconjunctivitis in Switzerland, showed significant differences with incidence peaks in autumn, while the fewest cases were reported in the summer months. Demographic data revealed a significant overrepresentation of male patients across both testing modalities.

The analysis of the COVID-19 period showed significant reduction in the observed number of infections, which might be associated with the effectiveness of hygiene measures in preventing infections with Adenovirus keratoconjunctivitis. Incidence rates quickly returned to pre-pandemic levels after cessation of quarantine measures. Further studies might be aimed at the inclusion of a wider range of medical centres to attain more reliable epidemiological data throughout the Swiss population, as well to include more data about clinical outcomes of the disease.

## Figures and Tables

**Table 1:** Overview of demographic characteristics of Adenovirus keratoconjunctivitis from May 2012 until March 2025.

Adenovirus	Assays Performed (*n*)	Positive Assays (% of All Tests)	Median Age (Years)	IQR Age (25th–75th Percentile)	Male (%)	Female (%)
All tests	747	179 (23.9)	40	31–54	58.1	41.9
PCR	557	128 (22.9)	40	32–54	58.6	41.4
AdenoPlus^®^	190	51 (26.8)	44	27–55	56.9	43.1

**Table 2:** Incidence rates of Adenovirus keratoconjunctivitis from May 2012 until March 2025, compared with the months during the COVID-19 pandemic (March 2020–February 2022).

	Complete Time Period (156 Months)	During COVID-19 (23 Months)	Outside of COVID-19 (133 Months)	Kruskal–Wallis Test (During COVID-19 vs. Outside COVID-19
Mean cases/month (SD)	1.15 (±1.27) *n* = 179	0.57 (±0.79) *n* = 13	1.25 (±1.31) *n* = 166	*p* = 0.009
Median (min, max)	1 (0.00; 6.00)	1 (0.00; 2.00)	1 (0.00; 6.00)	

**Table 3:** Incidence rates of Adenovirus keratoconjunctivitis from May 2012 until March 2025, comparing pre-COVID (May 2012–February 2020) with post-COVID (March 2022–March 2025) monthly infections. No statistical difference in incidence rates was found.

	Pre-COVID-19 (96 Months)	Post-COVID-19 (37 Months)	Kruskal–Wallis Test (Pre-COVID-19 vs. Post-COVID-19)
Mean cases/month (SD)	1.21 (±1.30) N = 96	1.35 (±1.36) N = 50	*p* = 0.465

## Data Availability

The original contributions presented in this study are included in the article. Further inquiries can be directed to the corresponding author.

## Abbreviations

The following abbreviations are used in this manuscript:

COVID-19	Coronavirus disease 2019
SARS-CoV-2	Severe acute respiratory syndrome coronavirus 2
PCR	Polymerase chain reaction
